# Inhibition of NTF4 Attenuates High Glucose‐Induced Apoptosis and Inflammation in HTR‐8/SVneo Cells via the PI3K/AKT Pathway

**DOI:** 10.1002/iid3.70460

**Published:** 2026-04-26

**Authors:** Li Zhang, Juan Yang

**Affiliations:** ^1^ Department of Obstetrics and Gynecology, The Central Hospital of Wuhan, Tongji Medical College Huazhong University of Science and Technology Wuhan China; ^2^ Key Laboratory for Molecular Diagnosis of Hubei Province, The Central Hospital of Wuhan, Tongji Medical College Huazhong University of Science and Technology Wuhan China

**Keywords:** apoptosis, gestational diabetes mellitus (GDM), inflammation, NTF4, PI3K/AKT pathway

## Abstract

**Objective:**

This study aims to investigate the role of neurotrophin‐4 (NTF4) in gestational diabetes mellitus (GDM) and to elucidate the underlying mechanism by which NTF4 regulates high glucose‐induced apoptosis and inflammation in HTR‐8/SVneo cells via the PI3K/AKT signaling pathway.

**Methods:**

Transcriptomic analysis combined with public database screening revealed that NTF4 is significantly upregulated in placental tissues from GDM patients. In a high glucose‐induced HTR‐8/SVneo cell model, NTF4 was silenced using small interfering RNA to evaluate the effects on cell proliferation, apoptosis, and inflammatory responses. Cell proliferation was evaluated using the CCK‐8 assay, apoptosis by flow cytometry, inflammatory cytokine secretion by ELISA, and cell migration and invasion by Transwell assays. Western blotting was performed to detect protein levels. Additionally, the PI3K‐specific inhibitor LY294002 was used to determine the pathway dependence of NTF4‐mediated effects. Results: NTF4 is upregulated in GDM placentas and high glucose‐induced HTR‐8/SVneo cells. Knockdown of NTF4 significantly ameliorated high glucose‐induced cell damage by enhancing cell viability, suppressing apoptosis, and inflammation. Mechanistic investigations revealed that NTF4 contributes to cellular injury by activating the PI3K/AKT signaling pathway, while the PI3K inhibitor LY294002 further amplified the protective effects of NTF4 silencing.

**Conclusions:**

This study identified the role of NTF4 in promoting trophoblast injury by activating the PI3K/AKT signaling pathway in high‐glucose‐stimulated HTR‐8/SVneo cells, which provides a preliminary experimental basis for exploring its potential role in the pathogenesis of GDM.

## 1 Introduction

Gestational diabetes mellitus (GDM) is a common metabolic disorder during pregnancy, typically occurring in the second or third trimester [1]. It is characterized by increased insulin resistance and impaired glucose metabolism. In recent years, the incidence of GDM has risen markedly, largely due to the growing prevalence of advanced maternal age and obesity, posing a serious threat to maternal and fetal health [2]. Studies have shown that GDM not only elevates the risk of pregnancy‐related complications such as gestational hypertension, preterm birth, and macrosomia, but also significantly increases the long‐term risk of diabetes, obesity, and cardiovascular diseases in offspring [3]. Moreover, women with GDM are at a substantially higher risk of developing type 2 diabetes postpartum, making GDM an emerging global public health concern [4].

As the core organ of maternal‐fetal material exchange and endocrine regulation during pregnancy, placental dysfunction is considered to be one of the key factors in the pathogenesis of GDM [5, 6]. Studies have shown that a hyperglycemic environment significantly impairs the migration and invasion capabilities of placental trophoblast, induces apoptosis, and promotes inflammation, thereby compromising placental structure and function, and interfering with embryonic development and pregnancy maintenance [7, 8]. Therefore, clarifying the mechanism of trophoblast damage and its key regulatory factors under high glucose stimulation is of great significance for understanding the pathogenesis of GDM and finding potential therapeutic targets.

By integrating data from the Comparative Toxicogenomics Database (CTD), GeneCards, and Gene Expression Omnibus (GEO) databases, this study identified that neurotrophin‐4 (NTF4) is aberrantly expressed in GDM and is enriched in signaling pathways such as PI3K/AKT. NTF4, a member of the neurotrophin family, primarily functions in neural development, cellular differentiation, and the regulation of apoptosis [9, 10]. Previous studies have demonstrated that NTF4 is dysregulated in various diseases and is closely associated with cell survival, proliferation, and signal transduction [11]. However, the role of NTF4 in GDM‐related placental tissues or trophoblasts remains unclear and warrants further investigation.

The PI3K/AKT signaling pathway plays a crucial role in regulating cell proliferation, glucose metabolism, and insulin signal transduction [12, 13]. The abnormal activation of the PI3K/AKT pathway is considered to be closely related to the occurrence of GDM [14]. Studies have shown that the activity of the PI3K/AKT pathway in the placenta of GDM patients is enhanced, accompanied by abnormal expressions of various downstream factors such as ANGPTL4 [15]. The PI3K/AKT pathway may be involved in the pathogenesis of GDM by influencing placental and pancreatic function and the inflammatory response.

This study aims to investigate the role of NTF4 in high glucose‐induced HTR‐8/SVneo cells, thus providing theoretical insights for elucidating the pathogenesis of GDM and developing targeted therapeutic strategies.

## 2 Materials and Methods

### 2.1 Bioinformatic Analysis

The gene expression profile of NTF4 in GDM was analyzed using the publicly available GSE154414 dataset from the GEO database. This dataset includes placental tissue samples from four women with GDM and four healthy pregnant women. Raw expression data were downloaded and preprocessed using the R software. Differentially expressed genes (DEGs) between the GDM and control groups were identified using the limma package. Genes with |log₂ fold change| > 1.0 and adjusted *p*‐value < 0.05 (Benjamini‐Hochberg correction for multiple testing) were defined as DEGs. Candidate GDM‐associated genes were further screened by the CTD and GeneCards databases. KEGG pathway enrichment analysis was subsequently conducted using the DAVID database to explore potential signaling pathways involved in GDM pathogenesis.

### 2.2 Cell Culture and Treatment

Human trophoblast cell line HTR‐8/SVneo was purchased from the Cell Bank of the Chinese Academy of Sciences (Shanghai, China). Cells were cultured in RPMI‐1640 medium supplemented with 10% fetal bovine serum (FBS; Gibco, USA) and 1% penicillin‐streptomycin (Gibco, USA) at 37°C in a humidified 5% CO₂ incubator. All subsequent small interfering RNA (siRNA) transfection experiments and functional assays were conducted using cells at passages 5–10 to avoid passage‐dependent phenotypic variability and ensure experimental reproducibility.

For high‐glucose (HG) stimulation, cells were treated with 25 mM glucose for 24 h, while the control group was maintained in 5.5 mM glucose. To further assess the role of the PI3K/AKT pathway, cells were pretreated with the PI3K inhibitor LY294002 (20 μM, Selleck Chemicals, USA) for 1 h prior to HG stimulation.

### 2.3 Cell Transfection

siRNAs targeting NTF4 (si‐NTF4, cat no. sc‐42127) and control‐siRNA (si‐NC, cat no. sc‐37007) were obtained from Santa Cruz Biotechnology (USA). Cells were transfected with 0.2 µM si‐NTF4 or 1 µM si‐NC using Lipofectamine 3000 reagent (Invitrogen, USA) following the manufacturer's instructions. After 48 h, transfection efficiency was confirmed by Quantitative Real‐Time PCR (RT‐qPCR) and Western blotting.

### 2.4 RT‐qPCR

Total RNA was extracted using a Total RNA Extraction Reagent (YI FEI XUE Biotechnology, China), and cDNA was synthesized using HiScript III RT SuperMix for qPCR (+gDNA wiper) (Vazyme, China). RT‐qPCR was performed using ChamQ Universal SYBR qPCR Master Mix (Vazyme, China) on a CG Real Time PCR (Heal Force). GAPDH was used as the internal control, and relative gene expression was calculated using the 2^−ΔΔCt^ method. Primer sequences are listed in Table 1.

**Table 1:** The RT‐qPCR primer sequences.

Name	Sequence (5′–3′)	
Homo GAPDH	Sense	AGGTCGGAGTCAACGGATTT
	Antisense	TGACGGTGCCATGGAATTTG
Homo NTF4	Sense	AACCCCCACCCTCAACATTG
	Antisense	cacctgctgactcccgaaag

### 2.5 Western Blotting

Proteins were extracted using RIPA lysis buffer (Beyotime, China) containing protease and phosphatase inhibitors (New Cell & Molecular Biotechnology, China). Equal amounts of protein were resolved by SDS‐PAGE and transferred to PVDF membranes (Millipore, USA). After blocking with 5% non‐fat milk, membranes were incubated with primary antibodies against NTF4 (cat no. 12297‐1‐AP, dilution 1:1000, Proteintech), PI3K (cat no. AF6241, dilution 1:1000, Affinity), p‐PI3K (cat no. AF3241, dilution 1:1000, Affinity), AKT (cat no. 10176‐2‐AP, dilution 1:2000, Proteintech), p‐AKT(cat no. 66444‐1‐Ig, dilution 1:2000, Proteintech), Bax (cat no. 50599‐2‐Ig, dilution 1:2000, Proteintech), Bcl‐2 (cat no. 26593‐1‐AP, dilution 1:1000, Proteintech), Cleaved‐Caspase‐3 (cat no. 25128‐1‐AP, dilution 1:1000, Proteintech), and GAPDH (cat no. 60004‐1‐Ig, dilution 1:50000, Proteintech). The following day, membranes were washed three times with TBST and incubated with HRP‐conjugated secondary antibodies (cat no. 7076/7074, dilution 1:1000, Cell Signaling Technology, USA) at room temperature for 1 h. Protein signals were visualized using an enhanced chemiluminescence detection kit (Beyotime, China), and band intensities were quantified using ImageJ software. GAPDH was used as the internal control.

### 2.6 Cell Viability Assay

Cell viability was evaluated using the Cell Counting Kit‐8 (CCK‐8) Assay. Briefly, HTR‐8/SVneo cells were seeded in 96‐well plates at 5000 cells/well and incubated at 37°C with 5% CO₂. At 0, 24, 48, and 72 h, 10 μL of CCK‐8 solution (New Cell & Molecular Biotechnology, China) was added per well and incubated for 2 h. Absorbance was measured at 450 nm using a microplate reader (Diatek, USA).

### 2.7 Flow Cytometry Analysis of Apoptosis

Apoptosis was assessed using the Annexin V‐FITC/PI Apoptosis Detection Kit (Yeasen Biotechnology, China) according to the manufacturer's instructions. Briefly, treated HTR‐8/SVneo cells were harvested and washed twice with cold PBS. Cells were resuspended in 300 μL of 1× Binding Buffer. Annexin V‐FITC (5 μL) was added and incubated in the dark for 10 min, followed by 10 μL PI for 5 min. Flow cytometric analysis was performed within 1 h using a FACSCanto II flow cytometer (BD, USA), and data were analyzed with FlowJo software.

### 2.8 Transwell Migration and Invasion Assays

Cell migration and invasion were assessed using Transwell chambers with 8 μm pore size inserts (Corning, USA). For the migration assay, 200 μL of serum‐free cell suspension (1 × 10⁵ cells/mL) was added to the upper chamber. For the invasion assay, the upper chamber membranes were pre‐coated with Matrigel (BD Biosciences, USA) diluted 1:8 in serum‐free medium and allowed to polymerize at 37°C for 1 h before seeding the cells. The lower chambers were filled with 500 μL of complete medium containing 10% FBS as a chemoattractant. The chambers were incubated for 48 h at 37°C in a humidified incubator with 5% CO₂. After incubation, cells remaining on the upper surface of the membranes were carefully removed with a cotton swab. The membranes were washed with PBS, fixed in 4% paraformaldehyde for 20 min, and stained with 0.1% crystal violet (diluted 1:4 from 0.5% stock solution) for 10 min. Excess stain was rinsed off, and the cells that migrated or invaded to the lower surface were imaged and counted in at least five randomly selected fields under an inverted microscope.

### 2.9 Lactate Dehydrogenase (LDH) Release Assay

Cellular cytotoxicity was assessed by measuring LDH release using an LDH Activity Assay Kit (Elabscience, USA) following the manufacturer's instructions. Absorbance at 450 nm was measured using a microplate reader.

### 2.10 Enzyme‐Linked Immunosorbent Assay (ELISA)

The concentrations of IL‐1β, IL‐6, TNF‐α, and IFN‐γ in culture supernatants were quantified using human ELISA kits (Elabscience, USA) following the manufacturer's instructions. Absorbance at 450 nm was measured using a microplate reader.

### 2.11 Statistical Analysis

All experiments were performed with three independent biological replicates. Data are presented as mean ± standard deviation (SD). Statistical analysis was conducted using GraphPad Prism 8.0. Differences between groups were analyzed using Student's *t*‐test or one‐way ANOVA followed by Tukey's test. A value of *p* < 0.05 was considered statistically significant.

## 3 Results

### 3.1 NTF4 Is Upregulated in High Glucose‐Induced HTR‐8/SVneo Cells

**Figure 1:**
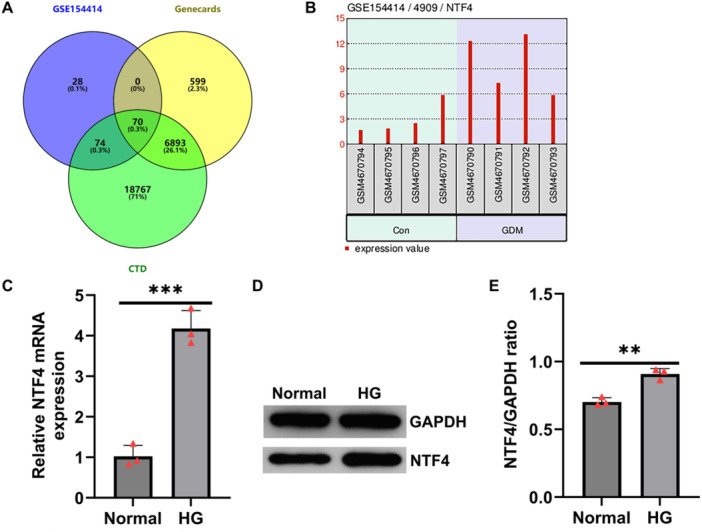
NTF4 is upregulated in HTR‐8/SVneo cells under high‐glucose conditions. (A) Venn diagram of 70 GDM‐related DEGs identified via GSE154414 and intersected with CTD and GeneCards databases; (B) NTF4 is significantly upregulated in GDM placental tissues (GSE154414); (C–E) RT‐qPCR and Western blotting confirm elevated mRNA and protein levels of NTF4 after 25 mM glucose treatment for 24 h. Data are presented as mean ± SD, *n* = 3. **p < 0.01; ***p < 0.001.

To investigate the expression profile and potential role of NTF4 in GDM, we integrated data from the CTD, GeneCards, and the GEO dataset GSE154414, identifying 70 DEGs significantly associated with GDM (Figure 1A). Among them, NTF4 was notably upregulated in placental tissues from GDM patients (Figure 1B), suggesting the potential involvement in the pathogenesis of GDM.

To validate this finding, HTR‐8/SVneo cells were exposed to 25 mM glucose for 24 h to establish an in vitro GDM model. RT‐qPCR revealed a significant increase in the mRNA level of NTF4 on HG stimulation (Figure 1C). Consistently, Western blotting demonstrated a marked elevation of NTF4 protein levels in the high glucose‐treated group compared to the control (Figure 1D,E). These results indicate that NTF4 is significantly upregulated in HTR‐8/SVneo cells under HG conditions.

### 3.2 Knockdown of NTF4 Alleviates High Glucose‐Induced Apoptosis and Improves Migration and Invasion in HTR‐8/SVneo Cells

**Figure 2:**
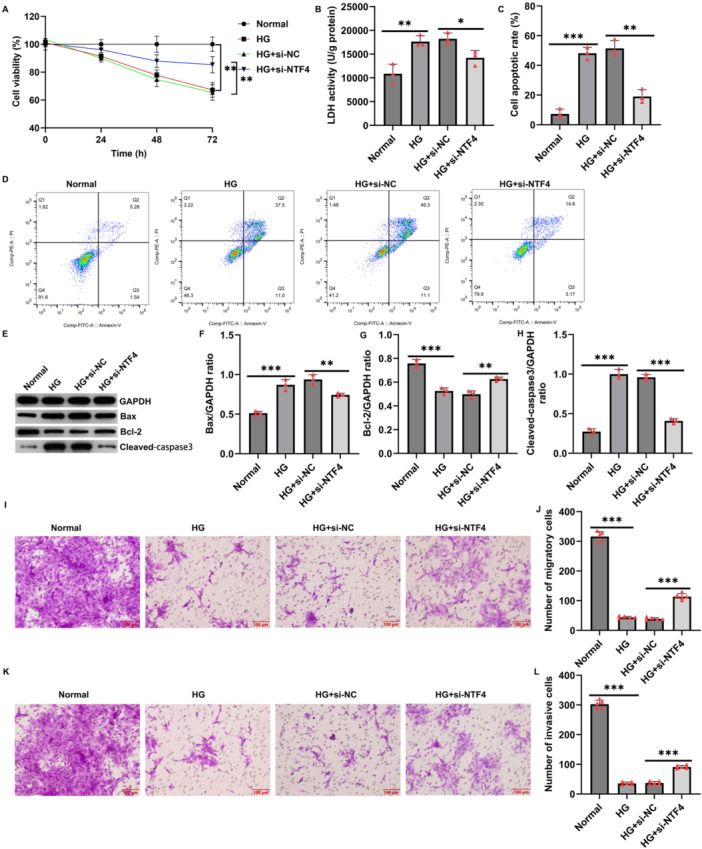
Knockdown of NTF4 Alleviates High Glucose‐Induced Apoptosis and Improves Migration and Invasion in HTR‐8/SVneo Cells. (A) CCK‐8 assay showed that NTF4 silencing restored cell viability suppressed by high glucose; (B) LDH activity was decreased by NTF4 silencing under high glucose conditions; (C and D) Flow cytometry revealed that NTF4 knockdown reduced high glucose‐induced apoptosis; (E–H) Western blotting demonstrated reduced Bax and cleaved caspase‐3 levels and increased Bcl‐2 following NTF4 knockdown; (I–L) Transwell assays showed that NTF4 knockdown improved migration and invasion impaired by high glucose. Data are expressed as mean ± SD, *n* = 3. *p < 0.05; **p < 0.01; ***p < 0.001.

To further investigate the role of NTF4 in high glucose‐induced trophoblast dysfunction, HTR‐8/SVneo cells were transfected with NTF4 siRNA (si‐NTF4) under high glucose conditions. After 48 h, RT‐qPCR and Western blotting confirmed that si‐NTF4 significantly reduced both mRNA and protein levels of NTF4 (Figure S1A–C). CCK‐8 assays revealed that high glucose treatment markedly suppressed cell viability in a time‐dependent manner, whereas NTF4 knockdown effectively rescued this suppression (Figure 2A). LDH release assays indicated increased cellular injury under HG conditions, which was significantly reduced following NTF4 knockdown (Figure 2B). Consistently, flow cytometry further demonstrated that high glucose exposure significantly increased apoptosis, which was notably alleviated by NTF4 silencing (Figure 2C,D). Western blotting revealed increased expression of pro‐apoptotic Bax and cleaved caspase‐3 and decreased anti‐apoptotic Bcl‐2 in the high glucose group, whereas NTF4 knockdown reversed these trends by downregulating Bax and cleaved caspase‐3 and upregulating Bcl‐2 (Figure 2E–H).

Transwell assays showed that high glucose markedly impaired the migratory and invasive capacities of HTR‐8/SVneo cells, while NTF4 silencing restored both functions (Figure 2I–L). These findings suggest that NTF4 knockdown effectively mitigates high glucose‐induced impairments in trophoblast viability, apoptosis, and motility.

### 3.3 Knockdown of NTF4 Attenuates High Glucose‐Induced Inflammatory Response in HTR‐8/SVneo Cells

**Figure 3:**
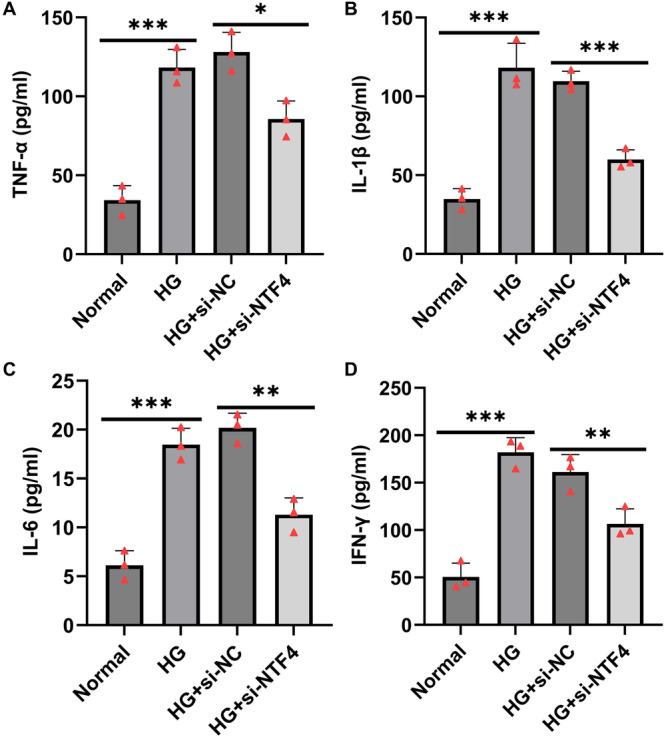
NTF4 knockdown alleviates high glucose‐induced inflammatory responses in HTR‐8/SVneo cells. (A–D) ELISA was used to detect the secretion levels of TNF‐α (A), IL‐1β (B), IL‐6 (C), and IFN‐γ (D) in the supernatants of cells in each group. Data are presented as mean ± SD, *n* = 3, *p < 0.05; **p < 0.01; ***p < 0.001.

To investigate the role of NTF4 in high glucose‐induced inflammation, HTR‐8/SVneo cells were transfected with si‐NTF4. The secretion levels of pro‐inflammatory cytokines TNF‐α, IL‐1β, IL‐6, and IFN‐γ in the cell culture supernatants were quantified using ELISA. Compared to the control group, high glucose exposure significantly increased the secretion of TNF‐α, IL‐1β, IL‐6, and IFN‐γ (Figure 3A–D). However, NTF4 knockdown under high glucose conditions markedly reduced the levels of IL‐1β, IL‐6, TNF‐α, and IFN‐γ (Figure 3A–D). These results suggest that NTF4 may contribute to HG‐induced inflammatory responses by promoting cytokine secretion, and its silencing effectively attenuates inflammation in trophoblast cells.

### 3.4 NTF4 Silencing Activates the PI3K/AKT Pathway in High Glucose‐Stimulated HTR‐8/SVneo Cells

**Figure 4:**
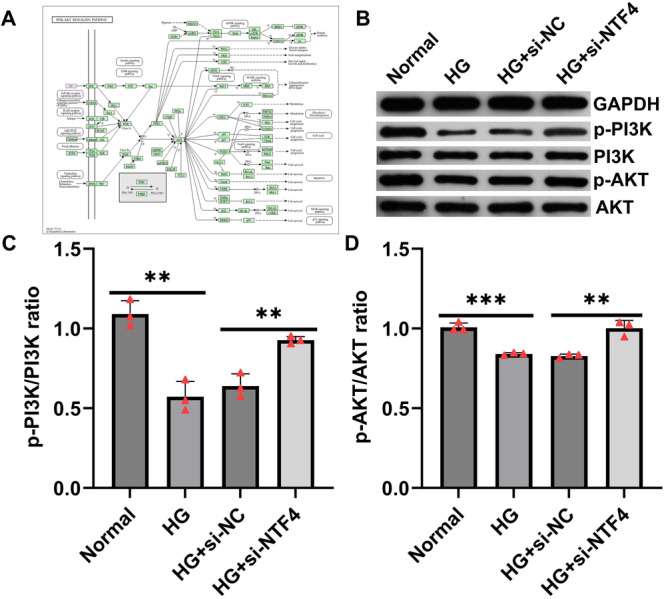
NTF4 silencing activates the PI3K/AKT signaling pathway in high glucose‐induced HTR‐8/SVneo cells. (A) KEGG pathway enrichment analysis shows that NTF4 was enriched in the PI3K/AKT signaling pathway; (B) Western blotting was performed to evaluate the expression and phosphorylation levels of key proteins in the PI3K/AKT signaling pathway in each group of cells; (C and D) Quantitative densitometric analysis showed that high‐glucose treatment markedly reduced the p‐PI3K/PI3K and p‐AKT/AKT ratios, while NTF4 knockdown significantly restored their phosphorylation levels. Data are presented as mean ± SD, *n* = 3. **p < 0.01; ***p < 0.001.

To further investigate the potential mechanism of NTF4 in GDM, KEGG pathway enrichment analysis was performed on the previously identified 70 DEGs associated with GDM. The results revealed significant enrichment in several signaling pathways related to cell survival and metabolism, including PI3K‐AKT and cGMP‐PKG pathways. Among them, the PI3K/AKT pathway was prominently enriched (Figure 4A), with NTF4 mapped within this regulatory network, suggesting the possible involvement in high glucose‐induced cellular dysfunction via this pathway.

To validate this prediction, we examined the expression of key proteins in the PI3K/AKT pathway. Western blotting showed that HG treatment markedly reduced the phosphorylation levels of PI3K and AKT, as evidenced by decreased p‐PI3K/PI3K and p‐AKT/AKT ratios compared to the control group (Figure 4B–D). Notably, silencing NTF4 under HG conditions significantly restored the phosphorylation of PI3K and AKT, indicating a reactivation of the pathway (Figure 4B–D). These findings suggest that NTF4 knockdown effectively activates the PI3K/AKT signaling pathway in high glucose‐stimulated HTR‐8/SVneo cells.

### 3.5 NTF4 Regulates High Glucose‐Induced Injury in HTR‐8/SVneo Cells via the PI3K/AKT Pathway

**Figure 5:**
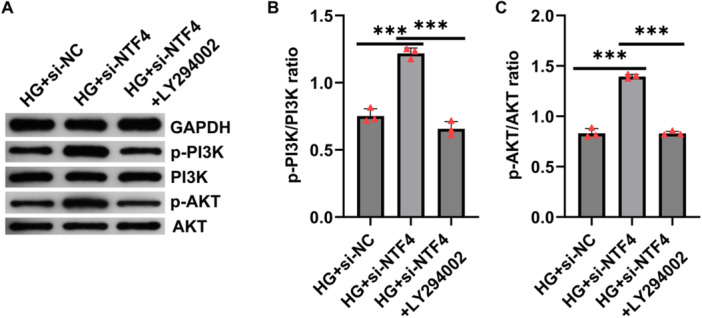
LY294002 reversed the activation of PI3K/AKT signaling pathway caused by NTF4 silencing in high glucose‐induced HTR‐8/SVneo cells. (A) Western blotting was performed to evaluate the expression and phosphorylation levels of key proteins in the PI3K/AKT signaling pathway in each group of cells. (B and C) Quantitative densitometric analysis showed that high‐glucose treatment markedly reduced the p‐PI3K/PI3K and p‐AKT/AKT ratios, while NTF4 knockdown significantly restored their phosphorylation levels. Data are presented as mean ± SD, *n* = 3. ***p < 0.001.

To elucidate the role of the PI3K/AKT signaling pathway in NTF4‐mediated cellular protection, we employed the PI3K‐specific inhibitor LY294002 in combination with NTF4 knockdown. At the signaling level, NTF4 knockdown notably increased the phosphorylation of PI3K and AKT, whereas LY294002 treatment significantly reduced their phosphorylation (Figure 5A,B).

**Figure 6:**
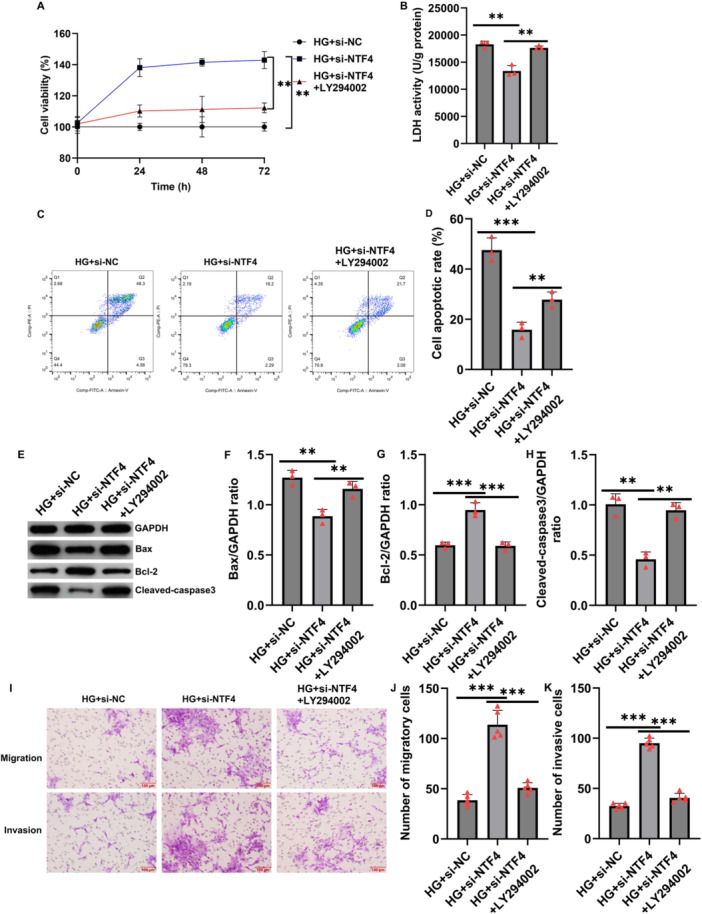
NTF4 modulates high glucose‐induced dysfunction in HTR‐8/SVneo cells via the PI3K/AKT pathway. (A) CCK‐8 assay to detect the proliferation of HTR‐8/SVneo cells; (B) LDH release assay; (C and D) Flow cytometry to detect the apoptosis of HTR‐8/SVneo cells; (E–H) Western blotting to detect the protein levels of Bax, Bcl‐2, and cleaved‐caspase3; (I–K) Cell migration and invasion ability was analyzed using Transwell assays; (F) ELISA assay was used to detect the secretion levels of IL‐1β, IL‐6, TNF‐α and IFN‐γ; (G) Western blotting to detect the protein levels of p‐PI3K and p‐AKT. Data are presented as mean ± SD, *n* = 3. **p < 0.01; ***p < 0.001.

CCK‐8 assays revealed that NTF4 silencing significantly enhanced the proliferation of HTR‐8/SVneo cells under HG conditions, whereas the addition of LY294002 markedly diminished this effect (Figure 6A). LDH release assays indicated that NTF4 silencing significantly reduced LDH levels, an effect reversed by LY294002 treatment (Figure 6B). Flow cytometry demonstrated that NTF4 knockdown significantly suppressed glucose‐induced apoptosis, which was reversed by LY294002 (Figure 6C,D). Western blotting further confirmed that si‐NTF4 reduced the expression of pro‐apoptotic Bax and upregulated anti‐apoptotic Bcl‐2, while PI3K inhibition restored Bax expression and decreased Bcl‐2 levels (Figure 6E–H). Transwell assays showed that NTF4 knockdown markedly promoted cell migration, whereas PI3K inhibition significantly impaired this functional recovery (Figure 6I–K).

**Figure 7:**
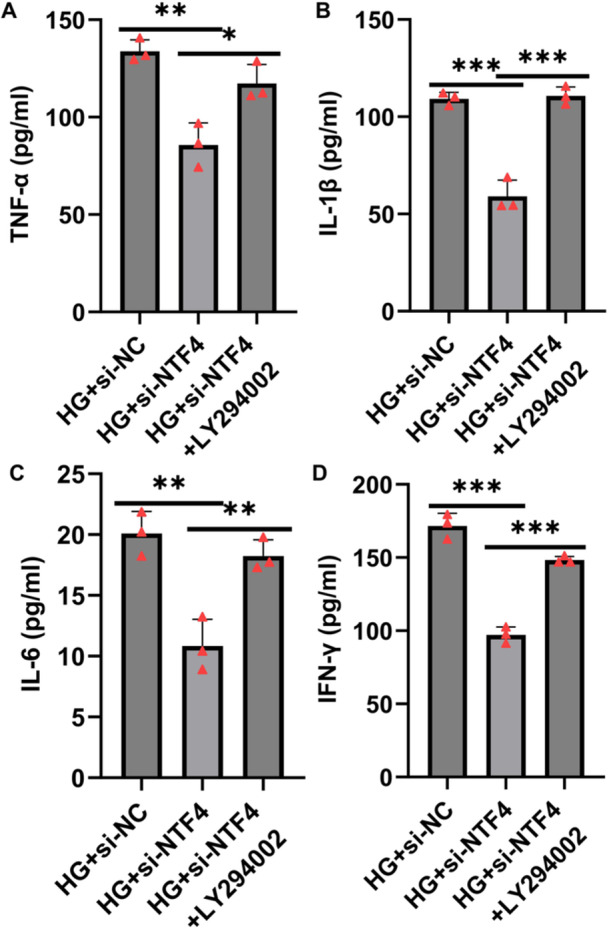
LY294002 reversed the suppression of the inflammatory response caused by NTF4 silencing in high glucose‐induced HTR‐8/SVneo cells. (A–D) ELISA was used to detect the secretion levels of TNF‐α (A), IL‐1β (B), IL‐6 (C), and IFN‐γ (D) in the supernatants of cells in each group. Data are presented as mean ± SD, *n* = 3. *p < 0.05; **p < 0.01; ***p < 0.001.

Furthermore, ELISA assays revealed that NTF4 silencing significantly suppressed the secretion of pro‐inflammatory cytokines including IL‐1β, IL‐6, TNF‐α, and IFN‐γ. However, LY294002 treatment reversed this anti‐inflammatory effect, leading to elevated cytokine levels (Figure 7A–D).

These findings suggest that NTF4 modulates high glucose‐induced injury by regulating the PI3K/AKT signaling pathway.

## 4 Discussion

GDM is one of the most common metabolic disorders during pregnancy, characterized by a complex etiology involving insulin resistance, chronic low‐grade inflammation, placental dysfunction, and programmed cell death [1]. In recent years, increasing attention has been directed toward the dysfunction of extravillous trophoblasts, which are considered a key contributor to GDM pathogenesis due to their heightened stress response under hyperglycemic conditions [16]. Therefore, elucidating the molecular mechanisms underlying trophoblast injury in an HG environment is of great clinical and scientific significance for understanding the pathological basis of GDM and identifying potential therapeutic targets.

In this study, we first identified that NTF4 is significantly upregulated in placental tissues from patients with GDM by integrating transcriptomic data with the GEO database. Consistently, expression of NTF4 was elevated in HTR‐8/SVneo cells exposed to HG conditions. Functional assays revealed that knockdown of NTF4 effectively alleviated high glucose‐induced trophoblast injury, as evidenced by enhanced cell viability, reduced apoptosis, and decreased secretion of pro‐inflammatory cytokines. Mechanistically, NTF4 was found to activate the PI3K/AKT signaling pathway. Importantly, treatment with the PI3K‐specific inhibitor LY294002 abrogated the protective effects of NTF4 silencing, indicating that the role of NTF4 is largely mediated through the PI3K/AKT pathway.

The PI3K/AKT signaling pathway is a classical cascade involved in the regulation of cell survival, apoptosis, and inflammatory responses, and plays a pivotal role in the progression of GDM [13, 17]. Aberrant activation of the PI3K/AKT pathway has been reported in various models of GDM. The PI3K/AKT pathway is critical for insulin signaling, and the dysregulation may impair GLUT4‐mediated glucose uptake, contributing to GDM development [18]. Ramenzoni et al. found upregulation of PI3K/AKT‐related genes in GDM placentas, suggesting that the abnormal activation may exacerbate metabolic stress and inflammatory responses in placental cells [19].

Recent studies have revealed that various transcription factors and microRNAs contribute to the pathogenesis of GDM by modulating the PI3K/AKT signaling pathway. For instance, placenta‐derived exosomal miR‐135a‐5p has been shown to promote trophoblast survival and exacerbate GDM progression by indirectly activating the PI3K/AKT pathway through targeting SIRT1 [20]. In contrast, miR‐22 and miR‐372 are consistently downregulated in patients with GDM and are thought to impair glucose metabolism by suppressing the PI3K/AKT/GLUT4 signaling axis [18]. Moreover, matrix metalloproteinases, such as MMP11 and MMP14, are markedly upregulated in the placentas of GDM patients and are closely associated with the activation of PI3K/AKT signaling, potentially contributing to abnormal placental remodeling and functional impairment [14]. Notably, natural bioactive compounds, such as ferulic acid glycosides, have also been found to ameliorate inflammation and apoptosis in GDM cell models by activating the PI3K/AKT pathway [21].

NTF4, a natural ligand of the TrkB receptor, was initially recognized for its critical role in the differentiation and repair processes of the nervous system. Previous studies have demonstrated that NTF4 can attenuate periventricular hemorrhage‐induced neuroinflammation in neonatal rats via activation of the TrkB/PI3K/FoxO1 signaling axis [22]. Additionally, NTF4 has been shown to induce the expression of myelin protein zero in Schwann cells through the TrkB/PI3K/Akt/mTORC1 pathway, thereby promoting myelination and neural regeneration [23]. In recent years, the functional roles of NTF4 have been increasingly identified in non‐neural tissues. For example, in breast cancer, NTF4 exhibits dual functions by participating in both tumorigenesis and the promotion of tumor cell migration and metastasis [24]. Moreover, NTF4 has been implicated in supporting the in vitro growth and maturation of human secondary follicles [25]. However, the role of NTF4 in pregnancy‐related disorders remains largely unexplored.

This study identifies NTF4 as a potential pro‐pathogenic factor in GDM, promoting high glucose‐induced apoptosis and inflammation via the PI3K/AKT pathway, but our experimental model has inherent limitations that require cautious interpretation of the findings. Hyperglycemia alone cannot recapitulate GDM's complex pathophysiology, which involves metabolic, hormonal, inflammatory, and oxidative stress perturbations, and the immortalized HTR‐8/SVneo cell line fails to reflect the heterogeneity of primary human trophoblasts and the complex cellular crosstalk in the in vivo placental microenvironment. Thus, our results are based on a homogeneous single‐factor in vitro model and cannot be directly translated to clinical GDM. Future studies will validate these findings in primary trophoblast cells from normal and GDM placentas, and in in vivo GDM animal models (streptozotocin‐ or high‐fat diet‐induced). Additional validation in distinct clinical GDM subtypes (insulin‐dependent/non‐insulin‐dependent) will further clarify NTF4's clinical applicability as a potential therapeutic target. Moreover, the precise mechanism by which NTF4 regulates the PI3K/AKT pathway (e.g., via TrkB or alternative mediators) and its role in other GDM‐related processes (glucose transport, angiogenesis, EMT) remain to be explored.

## 5 Conclusion

In conclusion, this study is the first to elucidate the critical role of NTF4 in the pathogenesis of GDM, demonstrating that NTF4 promotes high glucose‐induced trophoblast apoptosis and inflammatory responses by activating the PI3K/AKT signaling pathway. These findings provide a novel experimental perspective for understanding the molecular mechanisms of high glucose‐induced placental trophoblast dysfunction in GDM and preliminarily suggest that NTF4 may be a potential molecular target for GDM intervention, with its clinical translational value to be further verified in in vivo and clinical models.

## Author Contributions

**Li Zhang:** conceptualization, investigation, writing – original draft, methodology, data curation, supervision, project administration, formal analysis, software, visualization, resources. **Juan Yang:** data curation, writing – review and editing, writing – original draft. All authors have read and approved the final manuscript.

## Funding

The authors have nothing to report.

## Ethics Statement

The authors have nothing to report.

## Consent

The authors have nothing to report.

## Conflicts of Interest

The authors declare no conflicts of interest.

## Statement of AI Tools

No AI tools were used to write any part of this manuscript.

## Supporting information

Supporting File

## Data Availability

The datasets used and/or analyzed during the current study are available from the corresponding author on reasonable request.
