# Supplementary figures and images for: Inhibition of NTF4 Attenuates High Glucose‐Induced Apoptosis and Inflammation in HTR‐8/SVneo Cells via the PI3K/AKT Pathway
Source: Immun Inflamm Dis. 2026 Apr 26;14(4):e70460. doi: 10.1002/iid3.70460 (PMC13111406; doi:10.1002/iid3.70460)

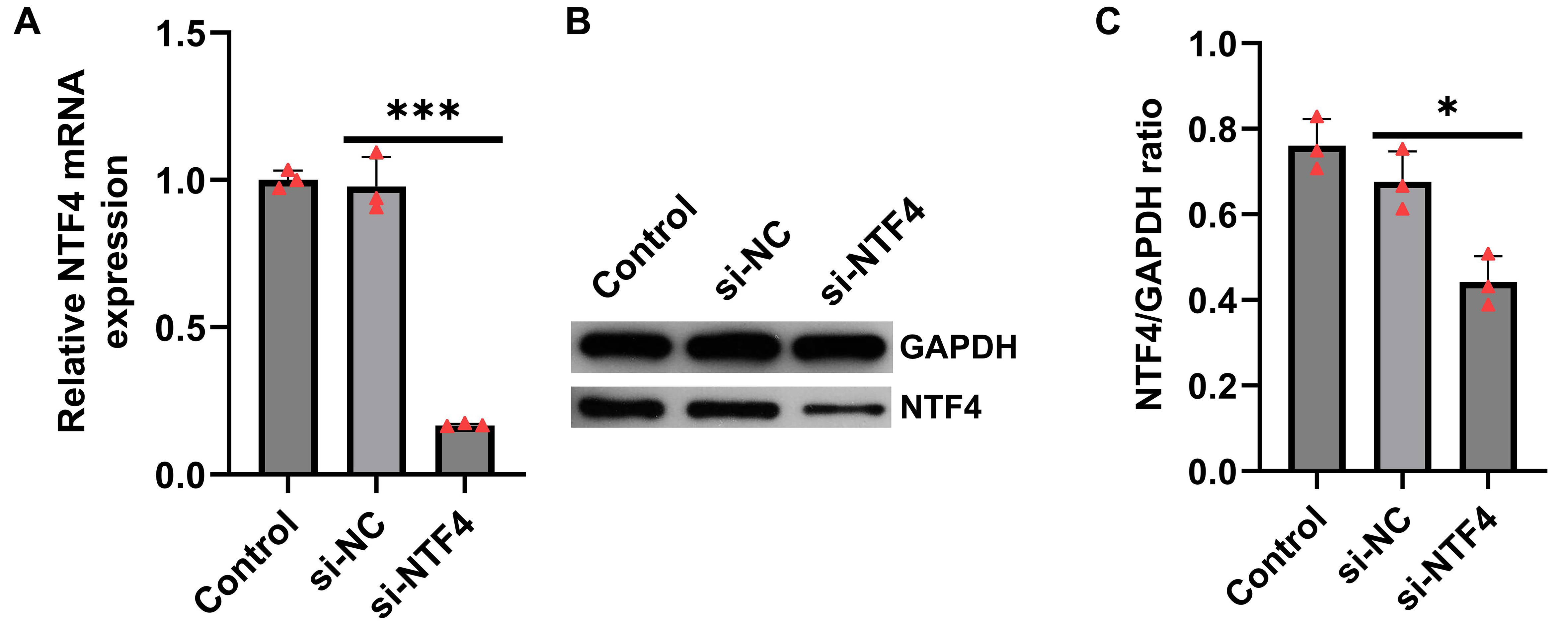

Supplement: Supplementary file 1 — Supporting File [file IID3-14-e70460-s001.jpg]
